# Loss-of-function mutations in the *CABLES1* gene are a novel cause of Cushing’s disease

**DOI:** 10.1530/ERC-17-0131

**Published:** 2017-05-22

**Authors:** Laura C Hernández-Ramírez, Ryhem Gam, Nuria Valdés, Maya B Lodish, Nathan Pankratz, Aurelio Balsalobre, Yves Gauthier, Fabio R Faucz, Giampaolo Trivellin, Prashant Chittiboina, John Lane, Denise M Kay, Aggeliki Dimopoulos, Stephan Gaillard, Mario Neou, Jérôme Bertherat, Guillaume Assié, Chiara Villa, James L Mills, Jacques Drouin, Constantine A Stratakis

**Affiliations:** 1 Section on Endocrinology and GeneticsEunice Kennedy Shriver National Institute of Child Health and Human Development (NICHD), National Institutes of Health (NIH), Bethesda, Maryland, USA; 2 Laboratoire de Génétique MoléculaireInstitut de Recherches Cliniques de Montréal (IRCM), Montréal, Québec, Canada; 3 Service of Endocrinology and NutritionHospital Universitario Central de Asturias, Instituto Universitario de Oncología del Principado de Asturias, Universidad de Oviedo, Oviedo, Spain; 4 Department of Laboratory Medicine and PathologyUniversity of Minnesota Medical School, Minneapolis, Minnesota, USA; 5 Surgical Neurology BranchNational Institute of Neurological Disorders and Stroke (NINDS), National Institutes of Health (NIH), Bethesda, Maryland, USA; 6 Newborn Screening ProgramWadsworth Center, New York State Department of Health, Albany, New York, USA; 7 Division of Intramural Population Health ResearchEpidemiology Branch, Eunice Kennedy Shriver National Institute of Child Health and Human Development (NICHD), National Institutes of Health (NIH), Bethesda, Maryland, USA; 8 Institut CochinINSERM U1016, CNRS UMR8104, Université Paris Descartes, Paris, France; 9 Department of NeurosurgeryHôpital Foch, Suresnes, France; 10 Service d’EndocrinologieCochin Hospital, Assistance Publique Hôpitaux de Paris, Paris, France; 11 Department of Pathological Cytology and AnatomyHôpital Foch, Suresnes, France; 12 Department of EndocrinologyCHU de Liège, University of Liège, Liège, Belgium

**Keywords:** Cushing’s disease, corticotropinoma, whole-exome sequencing, germline mutation

## Abstract

The *CABLES1* cell cycle regulator participates in the adrenal–pituitary negative feedback, and its expression is reduced in corticotropinomas, pituitary tumors with a largely unexplained genetic basis. We investigated the presence of *CABLES1* mutations/copy number variations (CNVs) and their associated clinical, histopathological and molecular features in patients with Cushing’s disease (CD). Samples from 146 pediatric (118 germline DNA only/28 germline and tumor DNA) and 35 adult (tumor DNA) CD patients were screened for *CABLES1* mutations. CNVs were assessed in 116 pediatric CD patients (87 germline DNA only/29 germline and tumor DNA). Four potentially pathogenic missense variants in *CABLES1* were identified, two in young adults (c.532G > A, p.E178K and c.718C > T, p.L240F) and two in children (c.935G > A, p.G312D and c.1388A > G, and p.D463G) with CD; no CNVs were found. The four variants affected residues within or close to the predicted cyclin-dependent kinase-3 (CDK3)-binding region of the *CABLES1* protein and impaired its ability to block cell growth in a mouse corticotropinoma cell line (AtT20/D16v-F2). The four patients had macroadenomas. We provide evidence for a role of *CABLES1* as a novel pituitary tumor-predisposing gene. Its function might link two of the main molecular mechanisms altered in corticotropinomas: the cyclin-dependent kinase/cyclin group of cell cycle regulators and the epidermal growth factor receptor signaling pathway. Further studies are needed to assess the prevalence of *CABLES1* mutations among patients with other types of pituitary adenomas and to elucidate the pituitary-specific functions of this gene.

## Introduction

In recent years, germline defects in multiple genes have been linked to the pathogenesis of pituitary adenomas, both with syndromic and isolated presentation (Caimari & Korbonits 2016). In contrast with other types of pituitary tumors, corticotropinomas are infrequent in patients with ‘classic’ multiple endocrine neoplasia and have rarely been described in the setting of familial isolated pituitary adenoma (FIPA) (Stratakis *et al.* 2010, Cazabat *et al.* 2012). Consequently, the germline abnormalities leading to corticotroph cell tumorigenesis remain largely unknown. On the other hand, somatic mutations in the *USP8* gene hotspot are highly prevalent among corticotropinomas in adult and pediatric patients, but they have not been detected in other types of pituitary adenomas (Pérez-Rivas *et al.* 2015, Reincke *et al.* 2015). Therefore, it might be that the majority of the corticotropinomas are caused by disruptions in molecular pathways that are not shared by other pituitary tumor types.

The *CABLES1* (Cdk5 and ABL enzyme substrate 1) gene (18q11.2) is a negative regulator of cell cycle progression that is activated in corticotroph cells in response to glucocorticoids (Roussel-Gervais *et al.* 2016). The physiological negative feedback exerted by glucocorticoids on the corticotroph cells is often impaired in corticotropinomas, and, concordantly, CABLES1 protein expression is lost in around half of such tumors (Roussel-Gervais *et al.* 2010, Roussel-Gervais *et al.* 2016). *CABLES1* gene inactivation by allelic loss, aberrant splicing or promoter hypermethylation has been observed in different types of human cancers but, to our knowledge, it has not been explored in pituitary adenomas before (Tan *et al.* 2003, Zhang *et al.* 2005, Sakamoto *et al.* 2008). Moreover, there are no human phenotypes reported in association with *CABLES1* germline mutations (http://omim.org/entry/609194, accessed: 28-03-17). Therefore, we investigated the presence of *CABLES1* gene mutations and copy number variations (CNV) in a large group of patients with Cushing’s disease (CD).

## Materials and methods

### Pediatric Cushing’s disease cohort

We studied 146 pediatric (<18 years at diagnosis) patients with CD who are part of a large cohort evaluated at the outpatient clinic and/or admitted for clinical work-up and treatment at the National Institutes of Health (NIH) Clinical Center between 1997 and 2017 and recruited under the research protocol 97-CH-0076 (ClinicalTrials.gov: Nbib1595). The *Eunice Kennedy Shriver* National Institute of Child Health and Human Development Institutional Review Board approved this study, and informed assent/consent was obtained from all the patients and their parents/guardians. Clinical data were obtained directly from the patients and/or from the Clinical Research Information System. Parents and siblings of the patients were also recruited, when available.

For all the individuals, DNA was extracted either from a peripheral blood sample using the Maxwell 16 Blood DNA Purification Kit in a Maxwell 16 Instrument (Promega AS1015 and AS3050) or from saliva using the Oragene-Dx collection kit and the PrepIT-L2P DNA extraction kit (DNA Genotek OGD-500 and PT-L2P-45), according to the manufacturer’s protocols. When available, stained histopathological sections from the corticotropinomas were retrieved from the Department of Pathology. After either manual delimitation of the tumor area or microdissection, DNA was extracted from unstained sections using the Pinpoint Slide DNA Isolation System (Zymo Research D3001). Screening for germline mutations in *AIP*, *CDKN1B*, *CDKN2C, MEN1*, and *PRKAR1A* in 74 of these patients, and *GPR101* in 34 of them, as well as somatic *GPR101* defects in 23 corticotropinoma DNA samples from these patients has been reported before (Stratakis *et al.* 2010, Trivellin *et al.* 2016).

Germline DNA samples from 98 patients and tumor DNA samples from 28 of them were submitted for whole-exome sequencing (WES) at the University of Minnesota Genomics Center. Targeted capture libraries were generated using the Agilent QXT v5 + UTRs kit for both germline and tumor samples. The germline samples were sequenced on an Illumina HiSeq 2000 platform producing 100 bp paired-end reads, while the tumor samples were sequenced on a HiSeq 2500 platform producing 125 bp paired-end reads. FASTQ files were processed using the steps delineated in the Broad Institute’s Genome Analysis Toolkit (GATK) best practices (Van der Auwera *et al.* 2013), including using BWA-MEM (Li & Durbin 2010) for alignment, GATK for quality recalibration and indel realignment and GATK HaplotypeCaller for genotyping. The median number of on-target reads generated per sample was 41 million, resulting in median target coverage of 54× (78% of targets covered at >20×). For *CABLES1*, the median percent of the 10 coding exons covered at 20× was 100% (average = 91%). The only exon with reduced coverage was exon 1, with 43% of samples having 20× coverage. ANNOVAR (Wang *et al.* 2010*a*) was used to determine the effect of coding variants using both the RefSeq and UCSC gene sets, including putative amino acid changes, distance to intron–exon boundary, the creation or removal of a stop-codon and location within known non-coding RNAs. Nonsynonymous variants were annotated based on their computationally predicted deleteriousness using information from dbNSFP. All variants were annotated for their frequency and presence in multiple variant collections (e.g., dbSNP, 1000 Genomes and internal WES datasets totaling over 10,000 samples). Targeted bioinformatic analysis ruled out rare mutations (present in <1% of any variant collection) in other known pituitary adenoma-associated genes (*AIP*, *CDKN1B*, *GPR101*, *MEN1* and *PRKAR1A*). For all the samples, a manual check of the WES raw data for *CABLES1* was performed, using the Integrative Genomics Viewer 2.3.72 platform (Broad Institute) (Robinson *et al.* 2011).

In addition, 48 other patients were screened for germline *CABLES1* variants by Sanger sequencing. The primers included in Supplementary Table 1 (see section on supplementary data given at the end of this article) were used to amplify the coding regions and exon–intron junctions by PCR (GoTaq Green Master Mix, Promega M7123) and for Sanger sequencing (BigDye Terminator 3.1 Cycle Sequencing Kit, Thermo Fisher Scientific 4337456). Sequences were analyzed using the SeqMan Pro 11.1.0 (DNASTAR) software. The Alamut Visual 2.9 software (Interactive Biosoftware) was used for the annotation, *in silico* prediction and to search the frequency in public databases of all the variants identified. Four algorithms (Align GVGD, PolyPhen-2, SIFT and Mutation Taster) were used for missense variants and five (Splice Site Finder, MaxEnt, NNSplice, GeneSplicer and Human Site Finder) were used for splicing variants. Variants were considered probably damaging or affecting splicing when the majority of the algorithms agreed; otherwise, they were considered variants of uncertain significance (VUS). Sanger sequencing confirmed all the variants identified by WES. Parents of the patients with *CABLES1* variants of interest were screened *ad hoc*, and loss of heterozygosity (LOH) was investigated in the corticotropinomas by Sanger sequencing.

### Collection of corticotropinomas from adult patients

Fresh frozen corticotropinoma tissue samples were obtained from 35 adult patients from Cochin Hospital and Foch Hospital operated for CD between 2009 and 2016. The local ethical committee approved the study, and all the patients provided written informed consent. RNA was extracted using the RNeasy kit (QIAGEN), and then reverse-transcribed. All the samples were submitted for RNA sequencing (RNA-seq) at the genomic platform of Cochin Institute. The libraries were prepared following the TruSeq mRNA protocol (Illumina RS-122-2101), starting from 1 μg of high-quality total RNA. Paired-end (2 × 75 bp) sequencing was performed in an Illumina NextSeq 500 instrument. Reads were aligned using the STAR software (Dobin & Gingeras 2015). Sequencing variants were annotated using ANNOVAR (Wang *et al.* 2010*a*) and then filtered for exonic nonsynonymous variants, with frequency <1% in the general population (based on the 1000 Genomes database) (1000 Genomes Project Consortium *et al.* 2015). Variants of interest were confirmed by Sanger sequencing in DNA extracted from representative sections of paraffin-embedded tissues, as described previously.

### Copy number variation analysis

CNV analysis was performed in 116 germline and 29 corticotropinoma DNA samples using FAM-labeled assays binding *CABLES1* exon 1, intron 3–exon 4 and exon 10 (TaqMan CNV assays Hs07536236_cn, Hs02003953_cn, and Hs00413958_cn, respectively, Thermo Fisher Scientific) a VIC-labeled *RPP30* (*Rnase P*) assay (Thermo Fisher Scientific 4403326) as an internal control, and the ddPCR SuperMix for Probes (no dUTP) (Bio-Rad 1863024) in a QX200 Droplet Digital PCR System (Bio-Rad). Results were analyzed with the Quanta Soft software 1.7.4.0917 (Bio-Rad).

### *USP8* screening

For patients with potentially pathogenic *CABLES1* variants, the primers 5′-CTTCCACCCCTCCAACTCAT-3′ and 5′-TGGAGTTACTGTTGGCTTCCT-3′ were used to amplify and sequence a region of 146 bp covering the *USP8* mutational hotspot in corticotropinoma DNA, as described above. Patients with *USP8* mutations from our cohort have been reported elsewhere (Pérez-Rivas *et al.* 2015, Faucz *et al.* 2017).

### Immunohistochemistry

In corticotropinomas from patients with putative *CABLES1* mutations and in two corticotropinomas with no mutations, immunohistochemical staining for ACTH (1:1000 rabbit polyclonal anti-ACTH antibody, Abcam 74976), CABLES1 (1:1000 rabbit polyclonal Abcam ab75535) and CDKN1B (1:100 rabbit polyclonal Santa Cruz sc-528) was performed as follows: deparaffinization for 30 min in Histo-Clear (National Diagnostics HS-200), sequential 5-min washes with 100, 95, 70 and 50% ethanol, antigen retrieval with citrate-based antigen unmasking solution (Vector H3300) for 20 min in a steamer, blocking for 1 h with 10% normal goat serum (Jackson ImmunoResearch Laboratories 005-000-121) in 1× PBS/0.1% Triton X-100, incubation with primary antibody overnight at 4°C, incubation with 1% H_2_O_2_ for 5 min, and then for 1 h with 1:1000 Biotin-SP AffiniPure Goat Anti-rabbit IgG and for 30 min with 1:500 Peroxidase Streptavidin (Jackson ImmunoResearch Laboratories 111-065-144 and 016-030-084, respectively). Samples were developed by incubation for 1 min with ImmPACT DAB peroxidase (HRP) substrate (Vector SK-4105) and counterstained with Gill’s hematoxylin I (American MasterTech Scientific HXGHE1PT) and Dako Bluing Buffer (Agilent Technologies CS70230-2). Sequential washes in a 50–100% ethanol gradient and Histo-Clear were done before mounting with Cytoseal XYL (Thermo Fisher Scientific 8312-4). Images were acquired using a Leica DMRX optical microscope, attached to an Olympus DP72 camera, and processed with the CellSens Dimension 1.6 software (Olympus).

### Expression plasmids

The mouse *Cables1* cDNA (variant 1: NM_001146287.1) was obtained from IDT and inserted downstream of the tamoxifen-inducible form of the estrogen receptor (ER_tam_) ligand-binding domain into a 3xFlag-tagged retroviral expression vector derived from pLNCX2 (Littlewood *et al.* 1995). Mutagenic primers were used to introduce the *Cables1* point mutations p.E139K (p.E178K in human, 5′-GCCACGAGTCCTCGGGAAACCCTCACAACCAC-3′ and 5′-GTGGTTGTGAGGGTTTCCCGAGGACTCGTGGC-3′), p.L201F (p.L240F in human, 5′-GGGTCAAGGGGTAGATTTAATTCCTTTACTCAGGG-3′ and 5′- CCCTGAGTAAAGGAATTAAATCTACCCCTTGACCC-3′), p.G273D (p.G312D in human, 5′-GTCGAACACTTTCAGATTCTCCTAGACCAAAG-3′ and 5′-CTTTGGTCTAGGAGAATCTGAAAGTGTTCGAC-3′) and p.D424G (p.D463G in human, 5′-GACCCAAACCTCCTGGGTGACCCCCAGTGGCC-3′ and 5′-GGCCACTGGGGGTCACCCAGGAGGTTTGGGTC-3′) in the plasmid, using the QuikChange site-directed mutagenesis kit (Agilent Technologies 200519) and the KOD hotstart DNA polymerase (Millipore 71086). The vectors were transfected into EcoPackTM 2-293 cells (Clontech 631506) to produce the viruses, which were then used to transduce AtT-20/D16v-F2 cells (ATCC CRL-1795).

### Cell culture, transfections and growth curves

The transformed AtT-20/D16v-F2 cells were grown in Dulbecco’s modified Eagle medium, supplemented with 10% fetal bovine serum and antibiotics. Pools of at least 1 × 10^3^ stable cell colonies were obtained as described (Rambaud *et al.* 2009) for pLNCX2-3xFlag-ER_tam_-Cables1 WT, pLNCX2-3xFlag-ER_tam_-Cables1 p.E178K, pLNCX2-3xFlag-ER_tam_-Cables1 p.L240F, pLNCX2-3xFlag-ER_tam_-Cables1 p.G312D, pLNCX2-Flag-ER_tam_-Cables1 p.D463G or pLNCX2-3xFlag-ER_tam_ empty vector as control. Cables1 protein levels were assessed by Western blot using 1 µg mouse monoclonal anti-Flag (SIGMA F1804) antibody. For growth curves, 7 × 10^5^ cells per well were plated in 6-well plates and treated in duplicate with 400 nM tamoxifen (SIGMA T5648), 10^−7^ M dexamethasone (SIGMA D4902), both or vehicle (100% ethanol). Viable cells were counted every 12–18 h for 4 days, starting 4 h after plating, and medium was replaced with fresh medium at every time point.

### Statistical analyses

All the analyses were carried out using the GraphPad Prism 7.0 software (GraphPad Software). Parametric data are presented as mean ± standard deviation (s.d.) and non-parametric data are presented as median ± interquartile range (IQR). Gene variant frequencies in the study population were compared with the frequencies reported in public databases using the Fisher’s exact test. For the cell growth experiment with WT *CABLES1*, cell counts at each time point were compared among experimental conditions using one-way ANOVA with Dunnett correction for multiple comparisons. For the rest of the experiments, cell counts at different time points were compared between ethanol and tamoxifen treatments using multiple *t* tests, with Holm–Sidak correction for multiple comparisons. Results were considered statistically significant when *P* < 0.05.

## Results

### Genetic findings

The pediatric CD cohort was composed of 72 males and 74 females, with mean age at first symptoms of 10.3 ± 3.3 years and age at diagnosis of 12.5 ± 3.4 years, for a median delay between disease onset and diagnosis of 26 months (IQR 12–38.3). The median pituitary tumor size was 4 mm (IQR 4–7); 88.1% (126/146) of the patients had microadenomas and 8.2% (12/146) had macroadenomas. Four patients (2.7%) had two or more adenomas and in four cases, the tumor was not identified, despite response to surgical treatment in three of them. The first line of treatment was transsphenoidal surgery in all the cases, except for one patient who developed spontaneous apoplexy of the pituitary adenoma. Ninety-seven percent of the patients (141/146) had sporadic presentation; among them, we identified two simplex patients with mutations in *AIP* and *PRKAR1A*, as previously reported (Stratakis *et al.* 2010, Hernández-Ramírez *et al.* 2017). Out of the five familial cases, three were members of families with a multiple endocrine neoplasia phenotype (two of them with *MEN1* mutations) as previously described (Stratakis *et al.* 2010) and two had a FIPA phenotype (family history of prolactinomas), but tested negative for *AIP* mutations. None of the patients had a family history of CD.

The adult cohort consisted of 35 CD patients, including 25 females and 10 males. Mean age at diagnosis was 44 years (range: 16–83). A microadenoma was found in 22.9% (8/35) and a macroadenoma in 77.1% (27/35) of the patients. Seventeen patients (48.6%) presented with overt CD, whereas clinical signs were limited or absent for the rest of them.

**Figure 1:**
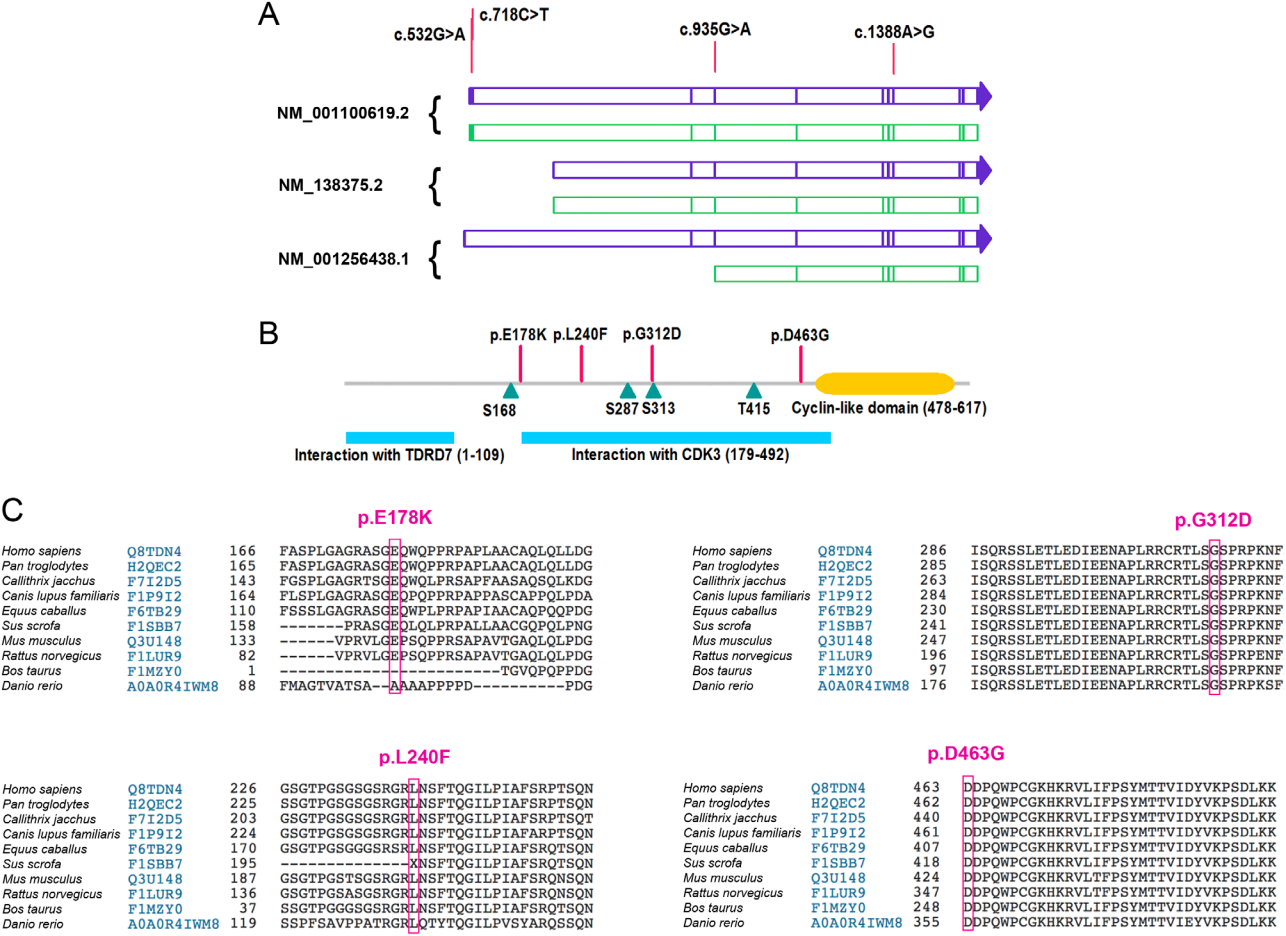
Missense CABLES1 variants of interest: effects on *CABLES1* transcripts and proteins. (A) Three transcripts of *CABLES1*, NM_001100619.2, NM_138375.2 and NM_001256438.1, which differ in their first exon, are translated into proteins of 633, 368 and 306 amino acids, respectively. The former (NM_001100619.2,) is the ‘canonical’ sequence (UniProt Q8TDN4-1). For each transcript, mRNA is schematized in blue and the coding sequence in green. The *CABLES1* variants identified in Patients 1 and 2 (p.E178K and p.L240F), respectively, affect exon 1 of the reference transcript, but not the other transcripts. The variant detected in Patient 3 is located in exon 3 (p.G312D) and the variant from Patient 4 (p.D463G) affects exon 7. There is no information in the literature about the expression of these transcripts in the pituitary gland. (B) The domains of the CABLES1 protein are incompletely characterized and no 3-D structure is available that represents the majority of the protein sequence. The 633 amino acid (65.7 kDa) isoform of the protein contains an N-terminal site for interaction with TDRD7 and a central large sequence necessary for interacting with CDK3. Four residues are targets for phosphorylation: S168, S287, S313 and T415 (Shi *et al.* 2015, Uniprot Consortium 2015, Yamochi *et al.* 2001). The C-terminal end of the protein contains a cyclin-like domain, and for this reason, CABLES1 has been included in the cyclin superfamily, although it differs in functions with cyclins. The alterations found in Patients 1–4 are included in (p.L240F, p.G312D, p.D463G) or very close to (p.E178K) the motif that interacts with CDK3. (C) The *CABLES1* gene is highly conserved among species, and the variants found in our patients affect relatively (p.E178K, p.L240F) and highly (p.G312D, p.D463G) conserved residues.

Four missense *CABLES1* single-nucleotide variants of interest (each in one patient) were identified among the 182 patients studied (Table 1), affecting exon 1 (c.532G > A, p.E178K and c.718C > T, p.L240F), exon 3 (c.935G > A, p.G312D) and exon 7 (c.1388A > G, p.D463G), respectively (Fig. 1 and Table 1). These missense variants were selected because of being either novel (p.D463G) or very infrequent in the general population (p.E178K, p.G312D) and/or because they were predicted to affect the protein structure (p.L240F, p.D463G). The ExAC database reported a frequency of 2.8% for the p.E178K variant: this might be an overestimation, since coverage for *CABLES1* exon 1 was low in the ExAC and gnomAD databases (Lek *et al.* 2016), while the 1000 Genomes database reported a much lower frequency. No pathogenic associations were reported for these variants in ClinVar or The Human Gene Mutation Database (Stenson *et al.* 2003, Landrum *et al.* 2016).

**Table 1:** Potentially pathogenic *CABLES1* variants identified in patients with Cushing’s disease.

							Control MAF (%)				P value				
**Patient ID (race/ethnicity)**	**DNA change (Ref Seq GRCH37/hg19: NM_001100619.2)**	**Protein change**	dbSNP ID	**Location in gene**	**Variant type**	**MAF among subjects in this study** (%)^a^	ExAC^b^	gnomAD^b^	1000 genomes	NHLBI EVS	MAF vs ExAC	MAF vs gnomAD	MAF vs 1000 genomes	MAF vs NHLBI EVS	*In silico* prediction
Patient 1 (Caucasian)	c.532G > A	p.E178K	rs200098768	Exon 1	Missense	0.2762	2.8436	0.9898	0.2796	n/a	0.0043^c^	ns	ns	n/a	Benign
Patient 2 (Caucasian)	c.718C > T	p.L240F	rs79793507	Exon 1	Missense	0.2762	0.0825	0.0783	0.2995	0.2799	ns	ns	ns	ns	Probably damaging
Patient 3^d^ (Hispanic)	c.935G > A	p.G312D	rs774334448	Exon 3	Missense	0.2762	0.0083 (0.0779)	0.0061 (0.0417)	n/a	n/a	0.0324 (ns)	0.0232 (ns)	n/a	n/a	VUS
Patient 4 (Hispanic)	c.1388A > G	p.D463G	NA	Exon 7	Missense	0.2762	n/a	n/a	n/a	n/a	n/a	n/a	n/a	n/a	Probably damaging

MAF, minor allele frequency; n/a, not available; ns, not significant; VUS, variant of uncertain significance.

a MAF among the screened patients. ^b^The frequency of variants located in the 5′ of the gene might not be accurate in the ExAC and gnomAD databases, as that region is poorly covered in those datasets. ^c^More common in ExAC than in our dataset. ^d^Population-specific MAF and *P* value of comparisons provided in parenthesis, when available.

In addition, other *CABLES1* variants (five of them not previously reported in public databases) were identified in the pediatric cohort (Supplementary Table 2). Given the methods used for screening, we could not accurately assess the frequency of intronic variants. No CNVs were found in the samples analyzed by ddPCR.

### Clinical, genetic and histopathological features in patients with putative *CABLES1* mutations

We identified putative *CABLES1* mutations in four sporadic female patients: two young adults (Table 2, cases 1 and 2) and two pediatric cases (cases 3 and 4). All these putative mutations were found in the heterozygous state in the germline DNA samples, and no LOH was detected in the pituitary adenomas. The four tumor samples were negative for *USP8* hotspot mutations. All these patients had large corticotropinomas (3/4 with extrasellar extension) with high Ki-67 proliferation index, and three of them required a second transsphenoidal surgery (and radiotherapy in one case) to achieve disease control.

**Table 2:** Patients with putative *CABLES1* mutations.

Patient ID	Cohort	***CABLES1* variant**	Gender	**Origin and ethnicity**	**Clinical presentation**	**Age at disease onset**	**Age at diagnosis**	Tumor size (mm)	**Ki-67 IHC** (%)	Treatment	**Disease status**
Patient 1	Adult CD	c.532G > A (p.E178K), heterozygous	F	French (Caucasian)	NFPA (silent corticotroph adenoma)	27	27	25	4	TSS (×2), RT	Stable residual tumor
Patient 2	Adult CD	c.718C > T (p.L240F), heterozygous	F	French (Caucasian)	Cushing’s disease	32	32	15	5	TSS	Unknown
Patient 3	Pediatric CD	c.935G > A (p.G312D), heterozygous (no LOH in tumor)	F	Guatemalan (Hispanic)	Cushing’s disease	15	16	19 × 11 × 14	>5	TSS (×2)	Active
Patient 4	Pediatric CD	c.1388A > G (p.D463G), heterozygous (no LOH in tumor)	F	Mexican-American (Hispanic)	Cushing’s disease	8	10	10 × 3 × 12	n/a	TSS	Remission

IHC, immunohistochemistry; n/a, not available; NFPA, non-functioning pituitary adenoma; RT, radiotherapy; TSS, transsphenoidal surgery.

Patient 1 presented at age 27 years with clinical manifestations due to the mass effect of her tumor (macroadenoma extending to the sphenoid and cavernous sinuses). She was diagnosed with a clinically non-functioning pituitary adenoma and two transsphenoidal surgeries followed by radiotherapy, were required to achieve control. Histopathological analysis established the diagnosis of a silent corticotroph adenoma, with microscopic invasion of the dura mater, bone and respiratory mucosa, Ki-67 index of 6% and 2 mitoses per 10 high-power fields.

Patient 2 presented with a clinical phenotype consistent with hypercortisolemia and was diagnosed with CD at age 32 years. Her pituitary macroadenoma with parasellar extension to the cavernous sinus was surgically removed. The resected tumor had a Ki-67 index of 8% and 7 mitoses per 10 high-power fields, but invasion of surrounding structures could not be assessed in the specimen. Since the patient was lost to follow-up, we have no data of her current status.

Patient 3 was diagnosed with CD at age 16 years, after a one-year history of hypertension, Cushingoid features and weight gain (weight: 131.4 kg, height: 160 cm, BMI: 51.3 kg/m^2^). She had elevated midnight cortisol (24.6 μg/dL), and urinary free cortisol (UFC, 437 μg/dL, ×11 ULN), unsuppressed serum cortisol (20.6 μg/dL) after a low-dose dexamethasone suppression test (LDDST) and her ACTH doubled during a CRH stimulation test (55.3–113 pg/mL). Her macroadenoma with suprasellar extension was resected, but a 15-month remission period was followed by recurrence, requiring a second surgery and remaining hypercortisolemic afterward. Genetic screening identified paternal inheritance of her putative *CABLES1* mutation, but despite being a carrier, her father was apparently unaffected.

Patient 4 had a congenital polycystic non-functional kidney, and her family history included one case of gastric cancer. She was evaluated at age 10 2/12 years because of decreased growth velocity (height 132 cm, −1 s.d.), hypertension, hirsutism and Cushingoid features, which developed in the previous 26 months. She had elevated ACTH (19.1 pg/mL) and midnight cortisol (19 μg/dL), failed a LDDST (cortisol 17 μg/dL) and had central hypothyroidism and dyslipidemia. Bilateral petrosal sinus sampling under CRH stimulation resulted in 3:1 central to peripheral ACTH ratio, confirming CD. Her pituitary adenoma without extrasellar extension was successfully resected, and the patient achieved remission. The *CABLES1* alteration found in the patient was absent in her mother’s DNA. As a sample from the father was not available, we cannot determine whether the variant was inherited or appeared *de novo*.

**Figure 2:**
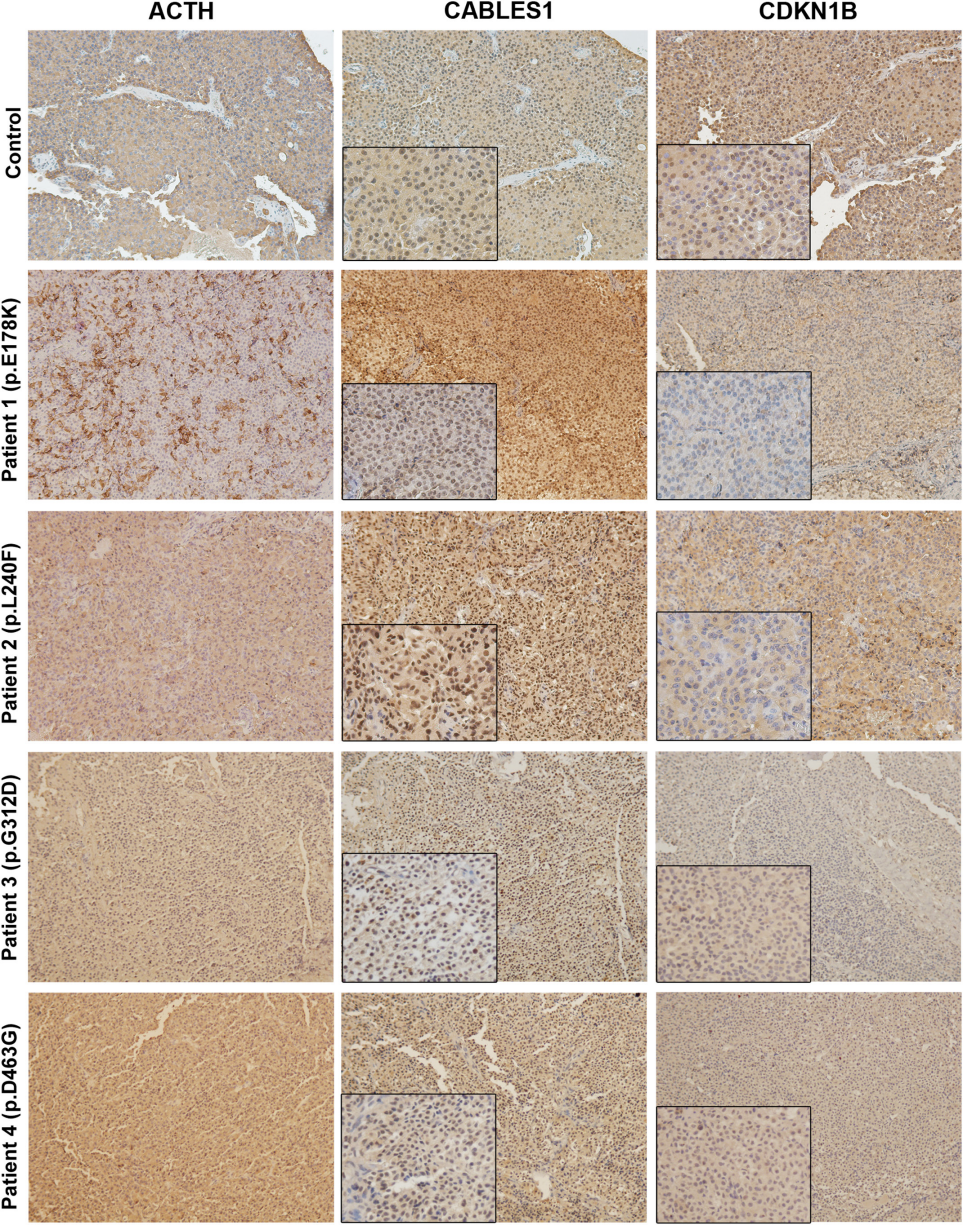
ACTH, CABLES1 and CDKN1B expression in corticotropinomas. We compared ACTH, CABLES1 and CDKN1B immunostaining in samples from the four patients with putative *CABLES1* mutations with samples from two patients negative for such alterations (one representative example is presented). ACTH immunostaining was observed in the vast majority of the cells in all the cases, except for Patient 1, for whom only 50% of the cells were immunoreactive (immunoreactivity for other pituitary hormones was ruled out). All the samples displayed positive CABLES1 cytoplasmic and, predominantly, nuclear immunoreactivity. In specimens that contained areas of non-tumoral pituitary, reduced CABLES1 staining was observed in the corticotropinomas, compared with the surrounding tissue (Supplementary Fig. 1), in concordance with previous data (Roussel-Gervais *et al.* 2016). Nevertheless, we did not observe differences in the CABLES1 immunostaining between samples with and without putative *CABLES1* mutations. In contrast, while in controls CDKN1B staining was moderately intense in the cytoplasm of the great majority of the cells and in the nucleus of 50–60% of them, only weak cytoplasmic staining and very few cells with nuclear staining were observed in the cases with putative *CABLES1* mutations (<10% cells with nuclear staining for Patients 1 and 2 and 30–40% cells with weak nuclear staining for Patients 3 and 4). Magnification in all the images: 10×, inserts: 20×.

Compared with samples without *CABLES1* alterations, samples from Patients 1 to 4 displayed reduced CDKN1B cytoplasmic and, more importantly, nuclear expression by immunohistochemistry (Fig. 2). No differences were found in the CABLES1 staining between samples with and without putative *CABLES1* mutations, although it was weaker in the tumors than in areas of non-tumoral pituitary (data not shown). This finding was not unexpected, as none of the samples displayed LOH for the variants identified, and points toward an alternative mechanism for reduced CABLES1 activity in the corticotroph cells.

### Functional assessment of putative *CABLES1* mutations

**Figure 3:**
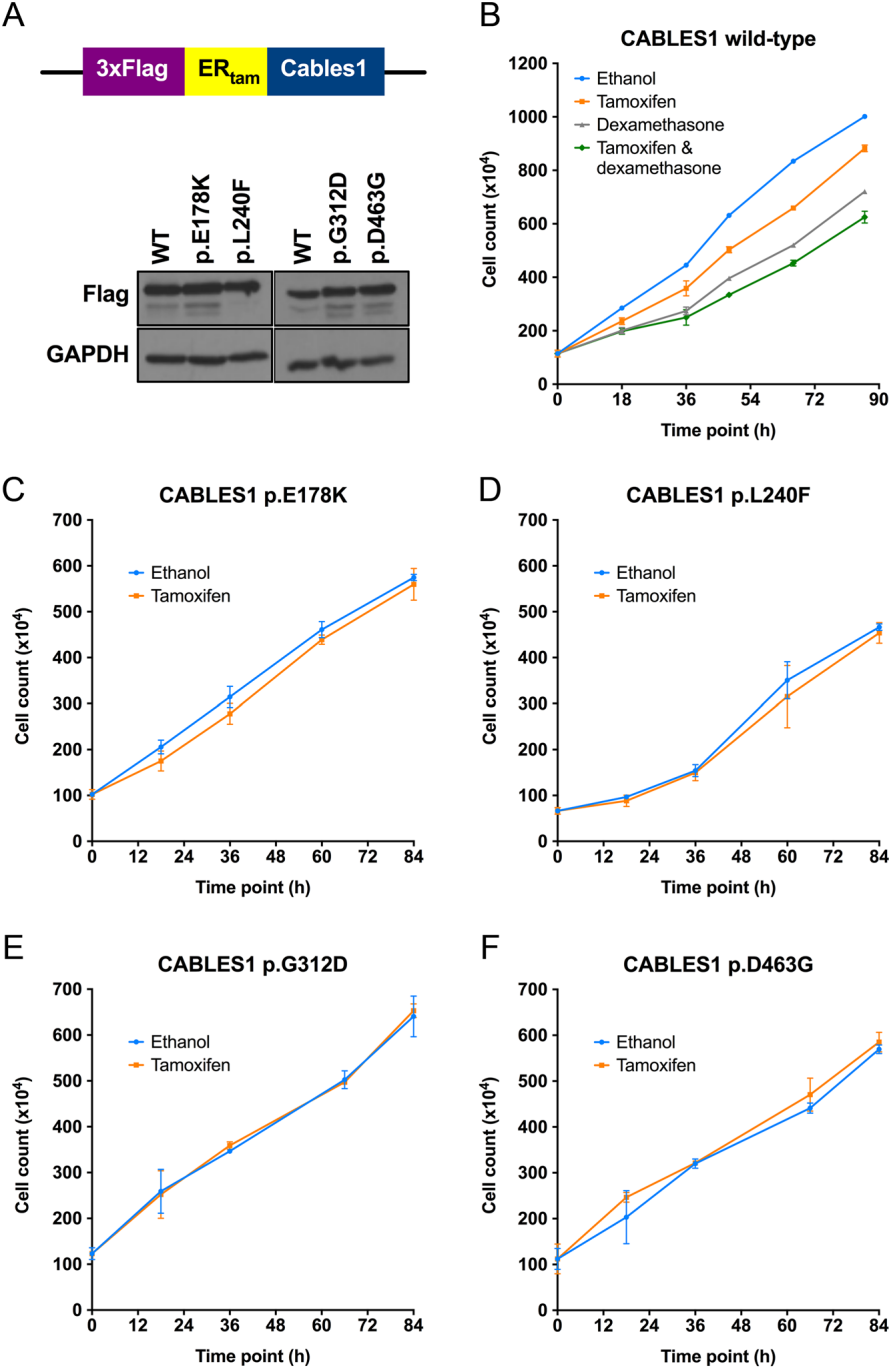
CABLES1 mutant proteins have lost their growth inhibition activity. (A) Schematic representation of tamoxifen-inducible chimeric CABLES1 proteins fused to the ER_tam_ ligand-binding domain. Upon stable transduction of AtT-20 cells, wild-type (WT) and mutant ER_tam_-CABLES1 proteins are expressed at similar levels as revealed by Western blot against the Flag epitope. (B) Growth curves of AtT20/D16v-F2 cells expressing WT ER_tam_-CABLES1 treated with vehicle (ethanol), tamoxifen, dexamethasone or both, as indicated. All the conditions rendered statistically significant differences in cell counts at all time points. To analyze exclusively the effect of CABLES1 on cell growth, only the tamoxifen treatment was used for experiments with the mutant proteins. (C, D, E and F) Growth curves for AtT20/D16v-F2 cells expressing CABLES1 p.E178K (C), p.L240F (D), p.G312D (E) or p.D463G (F) mutant protein in vehicle and tamoxifen-treated cells. There were no statistically significant differences in cell count at the different time points, meaning that these variants have lost the ability to suppress cell growth.

The conditional tamoxifen-inducible chimeric ER_tam_-CABLES1 proteins inserted by retroviral transduction were expressed at similar levels among the different AtT-20/D16v-F2 cell pools (Fig. 3A). As expected (Roussel-Gervais *et al*. 2016), WT CABLES1 inhibited the growth of AtT-20/D16v-F2 cells, but not as much as treatment with the synthetic glucocorticoid dexamethasone (10^−7^ M) or their combination (Fig. 3B). This result was expected, as AtT-20/D16v-F2 cells express *CABLES1* in response to dexamethasone, but *CABLES1* is only one of a group of dexamethasone-controlled cell cycle regulators (Roussel-Gervais *et al.* 2016). In contrast, p.E178K (Fig. 3C), p.L240F (Fig. 3D), p.G312D (Fig. 3E) and p.D463G (Fig. 3F) *CABLES1* mutants lost the ability to inhibit AtT-20/D16v-F2 cell growth. In summary, all the *CABLES1* variants identified in our patients affect a core property of *CABLES1*: its ability to inhibit cell growth in response to glucocorticoids.

## Discussion

The *CABLES1* gene has been recently identified as a key mediator of the regulatory feedback loop of glucocorticoids on the corticotroph cells (Roussel-Gervais *et al.* 2016). We have identified a small, but not negligible number (2.2%, 4/181) of patients with *CABLES1* alterations. The four putative mutations identified in our patients displayed reduced ability to block corticotroph cell proliferation in response to dexamethasone stimulation, supporting a role for *CABLES1* as a regulator of the corticotroph cell growth. Ours is the first study to identify a human phenotype associated with *CABLES1* germline mutations.

The CABLES1 protein was originally described as an interacting partner and substrate of the cyclin-dependent kinase-3 (CDK3) (Matsuoka *et al.* 2000, Yamochi *et al.* 2001). It plays an important role in neural development during embryogenesis, as inferred from *in vitro* studies and from the mouse and zebrafish models of gene inactivation (Zukerberg *et al.* 2000, Groeneweg *et al.* 2011, Mizuno *et al.* 2014). *CABLES1* is highly expressed in the nucleus of proliferating cells, where it is tightly regulated throughout the cell cycle, peaking at mid-late G1 (Matsuoka *et al.* 2000, Wu *et al.* 2001, Yamochi *et al.* 2001). In addition to CDK3, CABLES1 is a substrate for other protein kinases, including CDK2, 14-3-3, AKT, CDK5 and ABL, also serving as an adaptor protein for the last two (Zukerberg *et al.* 2000, Wu *et al.* 2001, Shi *et al.* 2015*b*). Another important function of this protein is to stabilize regulators of the cell cycle, such as CDKN1A (P21), CDK5R1 (P35) and TP63, preventing their degradation (Zukerberg *et al.* 2000, Wang *et al.* 2010*b*, Groeneweg *et al.* 2011, Shi *et al.* 2015*a*). CABLES1 also interacts with TP53 and TP73, triggering apoptosis (Tsuji *et al.* 2002).

The CABLES1 protein is widely expressed among tissues and is lost in a variety of human cancers (endometrial, ovarian, colorectal, lung and squamous cell carcinomas) (Kirley *et al.* 2005*a*). *CABLES1* gene inactivation promotes cell proliferation and survival, as well as tumor formation *in vitro* and replicates the human neoplasms in mouse models (Wu *et al.* 2001, Dong *et al.* 2003, Zukerberg *et al.* 2004, Kirley *et al.* 2005*a*,*b*). The tumor suppressor activity of *CABLES1* is inhibited by 14-3-3 or AKT-mediated phosphorylation (Shi *et al.* 2015*b*).

Interestingly, *CABLES1* could provide a link between two important molecular mechanisms disrupted in corticotropinomas: dysfunction of the CDK/cyclin-dependent cell cycle regulation and activation of the epidermal growth factor receptor (EGFR) pathway (Fukuoka *et al.* 2011, Liu *et al.* 2011*a*,*b*). The RAC-alpha serine/threonine-protein kinase (AKT1), one of the main effectors of EGFR, can inactivate CABLES1 by phosphorylation, neutralizing this way the regulatory effect of CABLES1 over the CDK/cyclin complexes (Fukuoka *et al.* 2011, Shi *et al.* 2015*b*).

Although a characteristic phenotype cannot be inferred from such a small number of patients, we found some noteworthy features. The four patients had young-onset macroadenomas, in agreement with previous observations suggesting that germline mutations in pituitary adenoma-causative genes are relatively frequent among patients with such characteristics (Stratakis *et al.* 2010, Tichomirowa *et al.* 2011). The tumors in the two adults were particularly large and aggressive, suggesting that, perhaps, they had arisen years before their diagnoses.

Considering that our patients had sporadic disease presentation, and given that corticotropinomas rarely remain asymptomatic over time, we could assume an incomplete penetrance for *CABLES1*-associated CD, which could explain the low frequency of these cases. Along these lines, it is important to emphasize that incomplete penetrance is a frequent finding among kindreds with pituitary adenoma-associated germline alterations (Williams *et al.* 2014, Iacovazzo *et al.* 2017). The study of large pedigrees with multiple cases of CD might be necessary to identify additional cases. However, the finding of multiple patients with CD in the same family is quite an infrequent phenotype: only 7.9% of the *AIP* mutation-negative FIPA families include CD, and only 1.4% of the families are ‘homogeneous’ for corticotropinomas (Hernández-Ramírez *et al.* 2015). On the other hand, as we could not establish the pattern of inheritance in three of the cases, *de novo* mutations cannot be confirmed or ruled out. A caveat of our study is that we could not rule out that the *CABLES1* mutations found in the adult patients were somatic defects, as germline DNA samples were not available from these cases. It is however noteworthy that the father of Patient 3 carries the *CABLES1* mutation: this may suggest that *CABLES1* mutations may predispose to CD and potentially be associated with very rare cases of familial CD, with incomplete penetrance.

Another interesting feature is the absence of somatic *USP8* mutations in our patients. This is the most common genetic defect so far identified in corticotropinomas, being found in around 40% of the tumors (Pérez-Rivas *et al.* 2015, Reincke *et al.* 2015). We have recently identified such mutations in one-third of our pediatric CD patients (Faucz *et al.* 2017). The absence of this somatic defect somehow mirrors the absence of *GNAS1* mutations in patients with somatotropinomas due to *AIP* mutations, suggesting that the genetic defects causing tumors due to germline predisposition are different than those found in non-inherited pituitary adenomas (Hernández-Ramírez *et al.* 2015).

The histopathological analysis of the samples revealed other common features among the four cases. CABLES1 expression ranged from low to moderate, but it was not completely absent in any of the tumors and was predominantly nuclear. However, the nuclear CDKN1B (P27) staining was particularly affected, compared with other corticotropinomas. Human germline *CDKN1B* mutations are associated with approximately 2% of the cases of *MEN1* mutation-negative multiple endocrine neoplasia (Georgitsi *et al.* 2007). Cases associated with *CDKN1B* mutations display a heterogeneous phenotype, referred to as MEN4, encompassing parathyroid and pituitary adenomas, neuroendocrine tumors and various benign and malignant tumors (Pellegata *et al.* 2006). Pituitary tumors have been reported in nine MEN4 patients so far, only one of them with CD (Agarwal *et al.* 2009, Lee & Pellegata 2013, Sambugaro *et al.* 2015). However, *Cdkn1b*-knockout (KO) mice develop, among other phenotypic abnormalities, ACTH-secreting hyperplasia or adenomas of the pituitary *pars intermedia* with full penetrance (Fero *et al.* 1996, Kiyokawa *et al.* 1996, Nakayama *et al.* 1996).

More importantly, nuclear CDKN1B immuno­staining is significantly reduced in pituitary adenomas of all types, particularly in corticotropinomas, in association with high Ki-67 expression, and is lost in pituitary carcinomas (Lidhar *et al.* 1999, Korbonits *et al.* 2002). This low expression is very likely not due to mutations or deletions, since these alterations have not been detected at the somatic level, but caused by posttranslational events (Dahia *et al.* 1998). The best-characterized mechanism for this in CD is increased CDKN1B phosphorylation by cyclin E (upregulated in corticotropinomas), which inactivates the protein and triggers its degradation, although 14-3-3 and AKT-mediated phosphorylation have the same effect (Korbonits *et al.* 2002, Susaki & Nakayama 2007, Roussel-Gervais *et al.* 2010). We observed that CDKN1B nuclear expression was particularly low in tumors with putative *CABLES1* mutations. As CABLES1 is known to stabilize and prevent the degradation of other cyclin-dependent kinases, we could speculate that our findings are due to increased CDKN1B degradation as a result of impaired CABLES1 function. This is in agreement with the rough correlation between CABLES1 and CDKN1B, but not cyclin E expression by immunohistochemistry in CD patients (Roussel-Gervais *et al.* 2016). Further experiments are required to confirm this mechanism, although they are beyond the scope of the present article.

We acknowledge the limitations of our study. Given that our centers are tertiary referral hospitals, aggressive or atypical cases of CD might be overrepresented in our cohorts. Although only a minority of the patients had macroadenomas, the frequency was higher than what is reported in the literature (Storr *et al.* 2011). The adult CD cohort included only patients for whom tissue was available, which means macroadenomas would be overrepresented. While these selection biases might preclude any inference on the prevalence of *CABLES1* variants in the general population of patients with CD, it was probably the uniqueness of our cases, which allowed us to identify such uncommon genetic alterations. Also, we do not have a firm basis to explain why the loss of function of a ‘generic’ tumor suppressor such as *CABLES1* caused a pituitary-specific phenotype in our patients. We could speculate that the missense variants found in these cases might cause loss of crucial pituitary-specific protein–protein interactions, but further studies are required to better characterize the function of this tumor suppressor in the corticotroph cells.

Our results provide evidence for a role of *CABLES1* as a novel pituitary tumor suppressor. The function of the CABLES1 protein as a regulator of different elements of the molecular pathways that control the cell cycle progression and apoptosis links genes previously known to have a role in the pathogenesis of CD. A more detailed characterization of such regulatory networks will allow a better understanding of the genetic basis of corticotropinomas, providing grounds for the search of novel therapeutic targets.

## Conclusions

We have identified four potentially pathogenic missense *CABLES1* variants as a novel, although infrequent, cause of CD in children and young adults. The CABLES1 protein functions as a glucocorticoid-responsive regulator of the effect of growth factor receptor activation on cell cycle control in the corticotroph cells. The putative *CABLES1* mutations found in our patients impair the ability of this tumor suppressor to control cell proliferation and might influence the function of CDKN1B by regulating its subcellular localization and/or degradation. Further studies are required to characterize in detail the function of this tumor suppressor in the corticotroph cells.

## Supplementary Material

Supporting Figure 1

Supporting Table 1

Supporting Table 2

## Declaration of interest

The authors declare that there is no conflict of interest that could be perceived as prejudicing the impartiality of this article.

## Funding

This work was supported by the Intramural Research Program, NICHD, NIH (DIR and DIPHR, NICHD and contract HHSN275201300023I), grants from the Canadian Institutes of Health Research (R G, A N, Y G and J D) and by the Fondation Foch (Foch Hospital, Suresnes, France) (C V).
