# Supplementary material for: Loss-of-function mutations in the CABLES1 gene are a novel cause of Cushing’s disease
Source: Endocr Relat Cancer. 2017 May 22;24(8):379–92. doi: 10.1530/ERC-17-0131 (PMC5510591; doi:10.1530/ERC-17-0131)
Supplement: Supporting Figure 1 [file erc-24-379-s001.pdf]

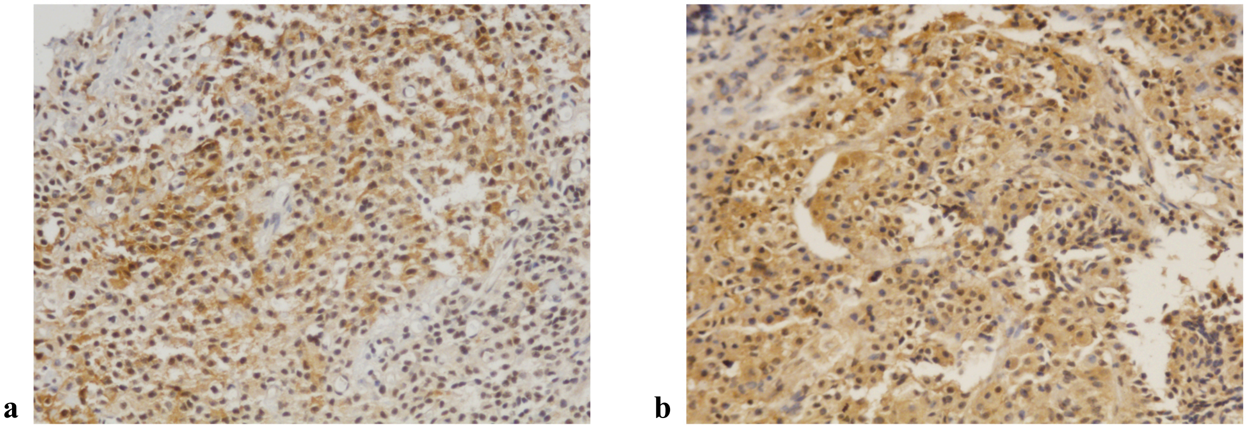

Supplemental Figure 1: CABLES1 immunostaining in non-tumoral pituitary tissue.
Representative images of non-tumoral tissue surrounding pituitary adenomas, a) Patient 3, b) Patient 4.
Magnification: 20X.
