# Supplementary material for: Loss-of-function mutations in the CABLES1 gene are a novel cause of Cushing’s disease
Source: Endocr Relat Cancer. 2017 May 22;24(8):379–92. doi: 10.1530/ERC-17-0131 (PMC5510591; doi:10.1530/ERC-17-0131)
Supplement: Supporting Table 1 [file erc-24-379-t001.pdf]

Supplemental Table 1: Primers for PCR and Sanger sequencing of *CABLES1*

Exon (NM_001100619.2)	Sequence (5'-3')	PCR product length (bp)
1	CTCGCTTCTCCGGGCATC	498
	AGGAATCAAGGGCGGCAG	
	GGTTCAGCTTGCTCGCCG	612
	GTGTCCCGTAGCCCAGAG	
2	TGCACAAATGTCTGATCAGTTCT	204
	ACCAAAATCAATCTCTGGCTGT	
3	AGACGTCTCAGCTGTCCAG	225
	AAAACTGTCCCCTCCTCCAG	
4	ATACCATCTGCCAGAGGAGG	292
	TGGCCACATTATGTACATTGGT	
5	GGAGTAAGCATCCTCATGGC	234
	CTCCTCTGCTTCCTGACCC	
6	AATGCAGAGCTAGCAATGCC	293
	GGAGACTCAATCCCCATGTAAA	
7	GCATTCTCCTGGCTGTCTC	277
	GGCAGAGCAGTCTTTGAACA	
8	GTTGGTCGTAGTTGGTGCAT	246
	TACGGTGTGTGGAAAGAGGG	
9	AATCCCAGTCCAGATCACCC	413
	TCACTGACCGAATCCTTCTGA	
10	GGTGGATAGAGAAGCTGCAATT	291
	AGTAGTAAGTAAGTGCTGCTCCA	
