# Supplementary material for: Loss-of-function mutations in the CABLES1 gene are a novel cause of Cushing’s disease
Source: Endocr Relat Cancer. 2017 May 22;24(8):379–92. doi: 10.1530/ERC-17-0131 (PMC5510591; doi:10.1530/ERC-17-0131)
Supplement: Supporting Table 2 [file erc-24-379-t002.pdf]

Supplemental Table 2: *CABLES1* variants identified in pediatric Cushing's disease patients

DNA change (Ref Seq GRCH37/hg19: NM_001100619.2)	Protein change	dbSNP ID	Location in gene	Variant type	Patient MAF (%)	Control MAF (%)				*P*-value*				*In silico* prediction
						ExAC	gnomAD	1000 Genomes	NHLBI EVS	MAF vs. ExAC	MAF vs. gnomAD	MAF vs. 1000 Genomes	MAF vs. NHLBI EVS	
c.-71G>A	p.(=)	rs113232639	upstream			n/a	36.6871	34.6845	n/a					no effects on splicing
c.225C>T	p.(=)	rs375018617	exon 1	synonymous	0.3425	0.5821	0.1579	0.0200	0.1938	ns	0.3763	ns	ns	no effects on splicing
c.295_303del	p.G99_A101del	rs139352344	exon 1	in-frame deletion	21.9178	62.0513	23.5397	22.4399	n/a	<0.0001***	ns	ns	n/a	no effects on splicing
**c.528G>T**	p.(=)	n/a	exon 1	synonymous	0.3425	n/a	n/a	n/a	n/a	n/a	n/a	n/a	n/a	no effects on splicing
c.567C>A	p.(=)	rs188544529	exon 1	synonymous	0.6849	1.4961	0.4784	1.1581	0.5707	ns	ns	ns	ns	creates a cryptic SA site
c.845+57C>T	p.?	rs142120848	intron 1	intronic		n/a	4.2828	4.9121	n/a					no effects on splicing
c.845+234C>T	p.?	rs4800451	intron 1	intronic		n/a	n/a	22.5639	n/a					activates a cryptic SA site
c.846-135T>C	p.?	rs80256063	intron 1	intronic		n/a	n/a	11.9609	n/a					no effects on splicing
c.866G>A	p.R289K	rs151062978	exon 2	missense	0.3425	0.1024	0.1216	0.2396	0.4353	ns	ns	ns	ns	VUS
c.917+65G>T	p.?	rs552048983	intron 2	intronic		n/a		0.1797	n/a					no effects on splicing
**c.917+83C>T**	p.?	rs768141252	intron 2	intronic		n/a		n/a	n/a					activates a cryptic SA site
**c.917+109A>G**	p.?	n/a	intron 2	intronic		n/a		n/a	n/a					no effects on splicing
c.917+176A>G	p.?	rs45464097	intron 2	intronic		n/a		19.5687	n/a					no effects on splicing
c.918-94A>G	p.?	rs2278453	intron 2	intronic		n/a		11.8011	n/a					no effects on splicing
c.918-44C>T	p.?	rs367731401	intron 2	intronic		0.0396		0.1797	0.0978					no effects on splicing
c.1011-153A>G	p.?	rs146254799	intron 3	intronic		n/a		1.0184	n/a					creates a cryptic SA site
**c.1011A>G**	p.(=)	n/a	exon 4	synonymous	0.3425	n/a	n/a	n/a	n/a	n/a	n/a	n/a	n/a	no effects on splicing
c.1065G>A	p.(=)	rs2304301	exon 4	synonymous	1.3699	2.6769	2.6511	3.8139	3.0454	ns	ns	<0.0342	ns	no effects on splicing
c.1088+211A>G	p.?	rs777609941	intron 4	intronic		n/a		n/a	n/a					no effects on splicing
c.1089-154A>G	p.?	rs2289012	intron 4	intronic		n/a		3.6342	n/a					no effects on splicing
c.1089-31C>T	p.?	rs7227728	intron 4	intronic		0.1963		0.4593	n/a					no effects on splicing
**c.1185+78C>A**	p.?	n/a	intron 5	intronic		n/a		n/a	n/a					no effects on splicing
c.1186-247C>T	p.?	rs117025081	intron 5	intronic		n/a		2.8355	n/a					activates a cryptic SA site
c.1186-144C>A	p.?	rs141394132	intron 5	intronic		n/a		0.8586	n/a					represses a cryptic SA site
c.1317C>T	p.(=)	rs35642798	exon 6	synonymous	1.0274	0.1475	0.1635	0.0599	0.2484	0.0019	0.0132	0.0030	ns	no effects on splicing
c.1343-164A>G	p.?	rs1966657	intron 6	intronic		n/a		13.0391	n/a					no effects on splicing

DNA change (Ref Seq GRCH37/hg19: NM_001100619.2)	Protein change	dbSNP ID	Location in gene	Variant type	Patient MAF (%)	Control MAF (%)				*P*-value*				*In silico* prediction
						ExAC	gnomAD	1000 Genomes	NHLBI EVS	MAF vs. ExAC	MAF vs. gnomAD	MAF vs. 1000 Genomes	MAF vs. NHLBI EVS	
c.1343-61C>A	p.?	rs45560835	intron 6	intronic		n/a		0.8586	n/a					no effects on splicing
c.1343-43G>A	p.?	rs45541339	intron 6	intronic		0.8647		0.8586	1.3960					no effects on splicing
c.1446+64C>A	p.?	rs2289013	intron 7	intronic		n/a		7.3682	n/a					no effects on splicing
c.1446+67G>C	p.?	rs12185467	intron 7	intronic		n/a		13.5383	n/a					no effects on splicing
c.1447-367G>A	p.?	rs6507568	intron 7	intronic		n/a		22.6637	n/a					no effects on splicing
c.1447-202T>A	p.?	rs2289014	intron 7	intronic		n/a		3.3347	n/a					activates a cryptic SD site
c.1447-105C>A	p.?	rs185106102	intron 7	intronic		n/a		0.1198	n/a					no effects on splicing
c.1553+59G>A	p.?	rs111634433	intron 8	intronic		n/a		3.3347	n/a					abolishes a cryptic SD site
c.1626C>T	p.(=)	rs201602276	exon 9	synonymous	0.3425	0.0832	0.0735	0.0599	n/a	ns	ns	ns	n/a	no effects on splicing
c.1740C>T	p.(=)	rs781259115		synonymous	0.3425	0.0008	0.0008	n/a	n/a	<0.0001	0.0036	n/a	n/a	no effects on splicing

* Comparison with public databases was done only for exonic variants. Only statistically significant values are shown.

** Alamut Visual v.2.9 was used to perfom *in silico* analyses. Four algorithms (Align GVGD, PolyPhen-2, SIFT, Mutation Taster) were used for missense variants, and five (Splice Site Finder, MaxEnt, NNSplice, GeneSplicer and Human Site Finder) were used for splicing variants. Variants were considered probably damaging or affecting splicing when the majority of algorithms agreed, otherwise they were considered VUS.

***More common in ExAC than in our dataset.

MAF: minor allele frequency; n/a: not available; ns: not significant; SA: splicing acceptor; SD: splicing donor; VUS: variant of uncertain significance. Bold: variant not reported in public databases.
